# One-Year Mortality Rates Before and After Implementing Quality-Improvement Initiatives to Prevent Inpatient Falls (2012–2016)

**DOI:** 10.3390/geriatrics3010009

**Published:** 2018-03-05

**Authors:** Inderpal Singh, Chris Edwards, Anser Anwar

**Affiliations:** 1 Department of Geriatric Medicine, Ysbyty Ystrad Fawr, Aneurin Bevan University Health Board, Ystrad Mynach, Wales CF82 7EP, UK; 2 Royal Gwent Hospital, Newport, Aneurin Bevan University Health Board, Wales NP20 2UB, UK; Chris.Edwards3@wales.nhs.uk; 3 Acute Medicine, Ysbyty Ystrad Fawr, Aneurin Bevan University Health Board, Ystrad Mynach, Wales CF82 7EP, UK; Anser.Anwar@wales.nhs.uk

**Keywords:** inpatient falls, death, hip fracture, hospital stay, single rooms

## Abstract

Single-room ward design has previously been associated with increased risk of inpatient falls and adverse outcomes. However, following quality initiatives, the incidence of inpatient falls has shown a sustained reduction. Benefits have also been observed in the reduction of hip fractures. However, one-year mortality trends have not been reported. The aim of this observational study is to report the trends in one-year mortality rates before and after implementing quality-improvement initiatives to prevent inpatient falls over the last 5 years (2012–2016). This retrospective observational study was conducted for all patients who had sustained an inpatient fall between January 2012 and December 2016. All the incident reports in DATIX patient-safety software which were completed for each inpatient fall were studied, and the clinical information was extracted from Clinical Work Station software. Mortality data were collected on all patients for a minimum of one year following the discharge from the hospital. The results show that 95% patients were admitted from their own homes; 1704 patients had experienced 3408 incidents of an inpatient fall over 5 years. The mean age of females (82.61 ± 10.34 years) was significantly higher than males (79.36 ± 10.14 years). Mean falls/patient = 2.0 ± 2.16, range 1–33). Mean hospital stay was 45.43 ± 41.42 days. Mean hospital stay to the first fall was 14.5 ± 20.79 days, and mean days to first fall prior to discharge was 30.8 ± 34.33 days. The results showed a significant and sustained reduction in the incidence of inpatient falls. There was a downward trend in the incidence of hip fractures over the last two years. There was no significant difference in the inpatient and 30-day mortality rate over the last five years. However, mortality trends appear to show a significant downward trend in both six-month and one-year mortality rates over the last two years following the implementation of quality initiatives to prevent inpatient falls. A significant reduction in the incidence of inpatient falls following quality initiatives initially has been observed, followed by a downward trend in the incidence of hip fractures. We have just started to observe a significant reduction in the 6-month and one-year mortality. We propose prompt completion of multifactorial falls risk assessments, and every possible quality initiative should be taken to prevent a ‘first inpatient fall’, which should result in the sustained improvement of clinical outcomes.

## 1. Introduction

The demographics of the human population are changing worldwide with a disproportionate increase in the oldest group. Hospitals are admitting a higher number of older people with acute illness. Hospitalisation is hazardous for frail older people and particularly for those with dementia [1,2]. The prevalence of falls increases with age, and the oldest groups are at highest risk. One-third of older adults over 65 years and half of older people above 80 years in the community could experience one fall in a year [3,4]. However, falls are more common among patients admitted to hospital. 

Hospital design may also influence the clinical outcomes of acutely ill, frail older people. New hospital-design policies favour single rooms over traditional multi-bed wards for greater privacy, personalised care and infection control. Single rooms previously have been associated with an increased risk of inpatient falls and adverse clinical outcomes [5]. Rates from 2.9 to 15.8 falls/1000 patient-bed days have been reported from different types of hospitals [5,6,7,8]. The rate of fall-related injury in Danish hospitals increased by an average of 11.4% annually between 2007 and 2012 [9]. Between 26.4% and 41.5% of inpatient falls may result in an injury or an associated adverse clinical outcome [7,10,11,12,13,14].

The National Audit of Inpatient Falls (NAIF), commissioned by the Healthcare Quality Improvement Partnership (HQIP) and managed by the Royal College of Physicians (RCP) UK, concluded that falls in hospital are the most commonly reported patient-safety incidents and remain a huge challenge for the National Health Service (NHS) [15]. More than 240,000 incidents of falls have been reported in acute hospitals and mental health trusts in England and Wales every year (that is over 600 incidents of falls per day) [15]. The NAIF recommends that all trusts and health boards have a falls steering group, falls multidisciplinary working group, and a review of multifactorial falls risk assessments (MFA). This should include all domains including dementia, delirium, blood pressure, a medication review, visual impairment, walking aids, a continence care plan, and call bells. These domains need to be linked to quality-improvement projects to ensure patient safety and quality [15]. 

Quality-improvement initiatives have been undertaken in a hospital under Aneurin Bevan University Health Board (UK) to minimise the incidence of inpatient falls [14]. The benefits have also been observed in the form of fewer fall-related complaints, some reduction in the length of stay, a reduction of inpatient hip fractures (IPHF) and discharge to original residence rather than a new care home. However, the impact of these quality initiatives on post-discharge one-year mortality after sustaining an inpatient fall during a hospital stay has not been reported. The aim of this observational study is to report the trends in one-year mortality rates before and after implementing quality-improvement initiatives to prevent inpatient falls over the last 5 years (2012–2016).

## 2. Methods

### 2.1. Study Design

This retrospective observational study was conducted to complete a descriptive analysis for all patients who have sustained an inpatient fall between January 2012 and December 2016 (5 years data). 

### 2.2. Setting

Ysbyty Ystrad Fawr (YYF) is a 269-bed local general NHS hospital in Ystrad Mynach, Wales, which was commissioned in 2011. YYF provides 100% single en-suite rooms and was designed in line with the current trend towards single rooms, which are thought to offer patients more control over their immediate environment and privacy and to minimise the spread of infection [16]. YYF admits acute and sub-acute patients and operates under the Aneurin Bevan University Health Board (ABUHB). 

### 2.3. Quality-Improvement Initiatives

Quality-improvement initiatives have been undertaken in a hospital with 100% single rooms with en-suite facilities to minimise the incidence of inpatient falls. The key mechanism for improvement was the education and training of nursing staff about fall risk factors. A plan–do–study–act (PDSA) methodology was used, and a geriatrician-led, systematic nurse-training programme on the understanding and correct use of existing multifactorial falls risk-assessment (FRA) tool was implemented in April 2013 [14]. The initial agreed action was to commence the ‘consultant-led weekly teaching’ and invite nursing staff from all the five medical wards to spread and drive the improvement. The nurses’ training programme was changed to once a fortnight after 3 months, and after 6 months a training session was conducted once a month until the end of 2015. Quality initiatives included systematic structured nursing training on falls to cover epidemiology and the impact of inpatient falls on patients, carers and organisation. Case-based discussion on common fall risk factors in the hospital, including dehydration, acute illness, delirium, dementia, appropriate footwear, accessibility to walking aids, continence promotion, nutritional assessment and the correct use of sensory aids was explored. A prompt completion of multi-factorial falls assessment and training was done to ensure that FRA is completed correctly, and particular emphasis was placed on the correct recording of postural hypotension and the polypharmacy review by doctors and pharmacists. Nurses were encouraged to ask for the previous history of fragility fractures and pursue medical teams to complete a bone health assessment. National and health board fall-prevention policies were also discussed with the intention of raising awareness about falls among healthcare assistants and providing enhanced physiotherapist intervention. These interventions have shown a sustained reduction of inpatient falls [14]. Falls training was conducted every two months but based on the feedback from the falls training programme, a sustained reduction of the inpatient fall incidence, and observed improved clinical outcomes; falls training is currently being conducted every 3 months to ensure sustainable improvement. 

### 2.4. Data and Measurements

Fall data were obtained from DATIX, a web-based patient safety software for reporting incidents. All the DATIX incident reports that are completed for each inpatient fall during admission to any of the five medical wards and medical admissions units were included. Incidents forms were excluded if it was not possible to extract patient details. The clinical information was extracted from Clinical Work Station software between January 2012 and December 2016. Data were transcribed, anonymised and incorporated into one password-protected Microsoft Excel sheet. 

To investigate the impact of inpatient fall, clinical outcome data were collected for individual patients including the length of stay (LOS), discharge destination, and inpatient fall-associated injury [17]. The mortality data was updated to the end of 31 December 2017. This was to ensure that mortality data has been recorded for a minimum of one year following discharge from the hospital for all patients who had sustained an inpatient fall until 31 December 2016

Sub-analyses for mortality as an inpatient, 30 days, 6-months and one-year post-discharge were completed to measure the trends in recorded mortality over the last five years (2012–2016). Sub-analyses for mortality were also done to measure its relation to a history of single inpatient fall versus recurrent (more than one) inpatient fall.

The index admission was defined as any single episode of admission until discharge from the health board or until death [17]. A fall in the hospital was defined using the same criteria as reported in the previous study [5]; an incident in which a patient came to rest on the floor or lower surface, without loss of consciousness. 

### 2.5. Statistical Analysis

All statistics were conducted using the STATISTICA StatSoft data analysis software system, version 9.1 (StatSoft, Inc., 2010, Tulsa, OK, USA). Means ± standard deviations were calculated for the baseline characteristics of all patients. Quality and patient-safety measures including regular systematic structured nurse training on falls and on the use of a multifactorial FRA tool were commenced in April 2013 [15]. Therefore, mortality analysis was compared before (2012–2014) and after (2015–2016) implementing quality-improvement initiatives to prevent inpatient falls. *t*-tests were used to compare the mean baseline characteristics of patients in both sites, and chi-square tests were used to compare categorical variables.

Mortality rates were compared using the difference between proportions test. Predictors of poor outcome (hip fracture and inpatient mortality) were explored using multivariate analysis for age, gender and number of falls. The generalised linear and non-linear models building module of the Statistica statistics package used binomial modelling with logit linking. *p*-values ≤ 0.05 were taken to be statistically significant. 

### 2.6. Ethical Approval

This study is classed as a service evaluation project according to the Health Research Authority decision tool. All documents to be used in data collection were submitted to the ABUHB Research and Development (R&D) department, which approved the project as an observational service evaluation with no requirement for ethical approval. All demographic and outcome data incorporated into the study are routinely documented by ABUHB and recorded in patient notes. Furthermore, no identifiable data besides sex, date of birth, and hospital identification number were recorded. No identifiable patient information has been or will be shared. 

## 3. Results 

The total number of incidents of falls reported between January 2012 and December 2016 (from 255,019 occupied-bed-days) was 3408, giving an overall falls incidence of 13.9/1000 occupied-bed-days; 104 (2.8%) incident reports were excluded from the analysis due to incomplete patient details. Data analysis was completed for 3304 inpatient-fall incidents, affecting 1704 admission episodes or index admissions. 

The mean age on admission for patients who sustained an inpatient fall was 81.04 ± 10.37 years. The proportion of females who sustained an inpatient fall was slightly higher (51.8%) but the mean age of females (82.61 ± 10.34 years) was significantly higher than of males (79.36 ± 10.14 years). Mean falls/patient were 2.0 ± 2.16 (range = 1–33). Mean hospital stay was 45.43 ± 41.42 days. Mean days to first fall from admission to hospital was 14.5 ± 20.79 days, and mean days to first fall prior to discharge was 30.8 ± 34.33 days. 

Of the patients who sustained an inpatient fall, 95% (*n* = 1619/1704) were previously living in their own homes and only 5% (*n* = 85/1704) were admitted from care homes; 66.8% patients (*n* = 924/1382) were discharged to their original residence, and 33% (*n* = 458/1382) were discharged to a care home; 373 patients required a new care-home placement, which represents an approximately 4.5 times higher rate of care-home occupancy than the patients being originally admitted from a care home. 

**Figure 1:**
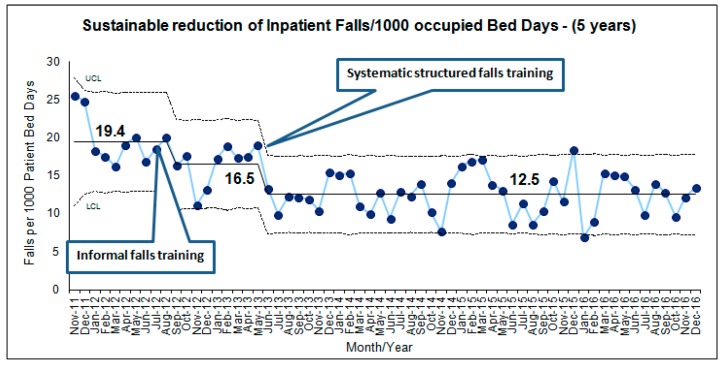
Statistical process U-control chart displaying sustainable reduction of the incidents of inpatient falls in a time sequence over 5 years.

The incidence of falls had shown a significant reduction (*p* < 0.001) with sustained effects (inpatient fall = 12.5/1000 occupied-bed-days) over the next 3.5 years during which quality initiatives were introduced (Figure 1). The most recent annualised falls incidence (2016) was 12.2 ± 2.72, which continues to show a sustainable reduction. However, the number of patients who sustained one or more falls in the hospital has remained the same over the last five years (2012–2016). The mean number of patients who sustained an inpatient fall was 340 and ranged from 336 to 348. 

**Figure 2:**
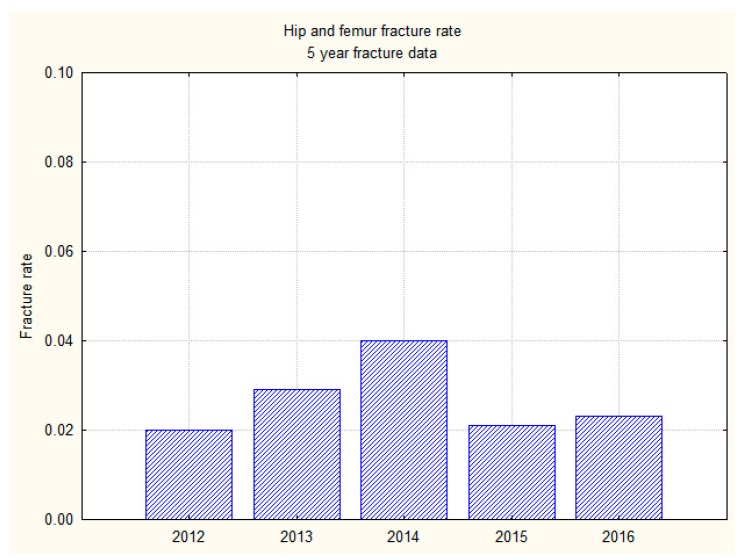
A bar graph showing hip fracture trend over 5 years (2012–2016).

The incidence of IPHF in this study over the last five years ranged from 2.0% to 4.0% (7–14 fractures/year). The mean hip fracture over the last five years was 2.7% (*n* = 46/1704). A downward trend in the incidence of hip fracture rate has been observed over the last two years, but it is not statistically significant. (Figure 2). 

**Figure 3:**
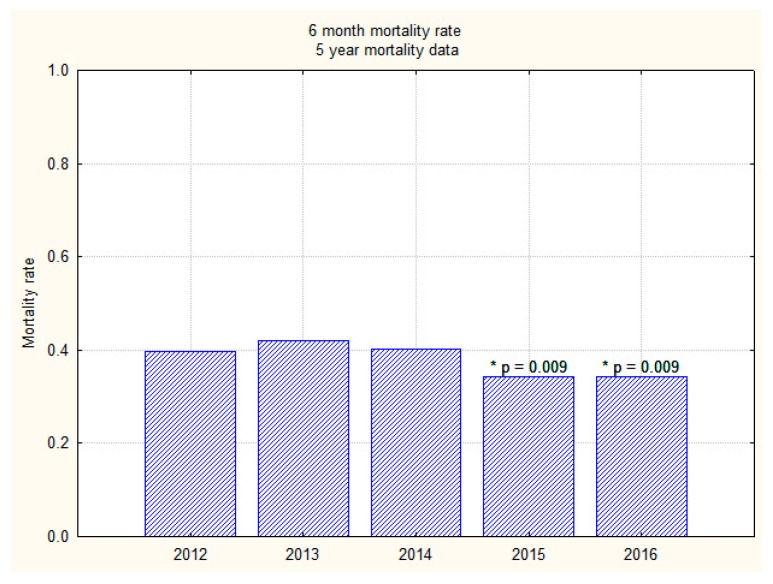
A bar graph showing 6-month mortality trend over 5 years (2012–2016). * A significant reduction (*p* < 0.01).

**Figure 4:**
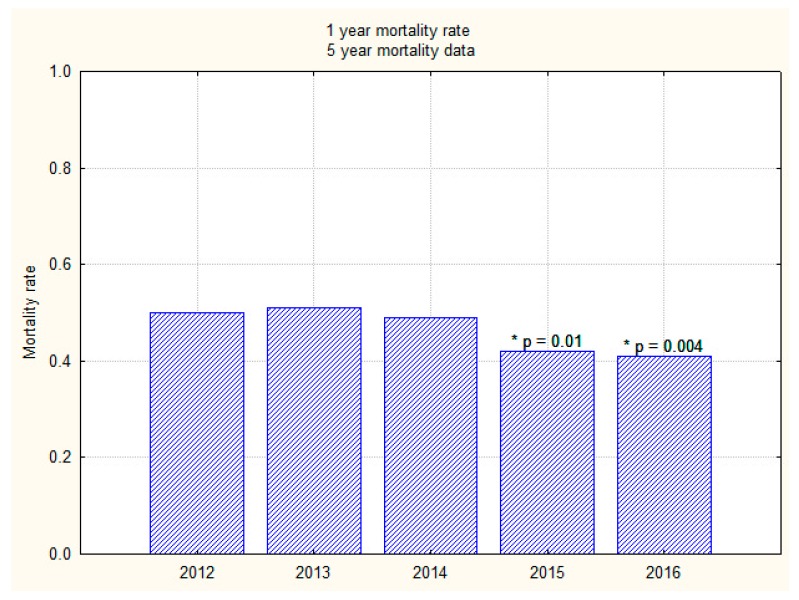
A bar graph showing one-year mortality trend over 5 years (2012–2016). * A significant reduction (*p* < 0.01).

Overall inpatient mortality for those who sustained an inpatient fall was 18.8% (*n* = 322/1704); 30-day and 6-months mortality were 23.7% (*n* = 404/1704) and 38.0% (*n* = 649/1704), respectively. Overall one-year mortality (2012–2016) was 46.4% (*n* = 792/1704). There was no significant difference in the inpatient and 30-day mortality over the last five years before and after intervention (2012–2016). However, mortality trends appear to show a significant downward trend in both the 6-month and one-year mortality rates over the last two years (2015 and 2016) as compared to the previous three years (2012–2014) (Figure 3 and Figure 4).

Sub-analysis for inpatient mortality was done for single or recurrent inpatient falls. Inpatient mortality for those with a single inpatient fall was 16.8% (*n* = 172/1022); in comparison, for those who sustained recurrent inpatient falls during an index admission, inpatient mortality was significantly higher (21.9%, *n* = 150/682) (*p* = 0.008, a difference in proportions test). 

Sub-analyses were also completed for patients who sustained injury during an index admission; 5.8% (*n* = 99/1704) patients (mean age = 82.1 ± 8.6 years, females = 58.6%) sustained a serious injury which included: hip fracture, 2.7% (*n* = 46/1704); non-hip fracture, 2.9% (*n* = 50/1704); and head injury, 0.2% (*n* = 3/1704). Of those who had IPHF, 70% of patients (*n* = 32/46) fractured following a ‘first inpatient fall’ and mean days to fall resulting in a hip fracture from admission to hospital were 18.59 ± 17.54 days (range = 1–86 days). The mean LOS for those who sustained IPHF was 53.80 ± 55.80 days; 28% (*n* = 13/46) patients died as inpatients; 33% (*n* = 15/46) required a new care home; and 39% of patients (*n* = 18/46) were discharged back to their original residence. Overall, one-year mortality following IPHF was 56.5% (*n* = 26/46). 

Sub-analyses were also done to establish predictors of poor outcome: hip fracture and inpatient mortality. Variables of age, gender and number of falls did not significantly predict hip fractures. Variables of age, gender and occurrence of hip fracture were found to be significant predictors of inpatient mortality (*p* < 0.001 for each variable). Each variable is a predictor of inpatient mortality, but on their own are not as strong as predictors as when they are combined.

## 4. Discussion

Inpatient falls are the commonest serious incident reported in a hospital setting and are associated with adverse clinical outcomes. IPHF constitutes a significant proportion of hip fractures and had a very poor clinical outcome including higher mortality and prolonged LOS [18,19]. The risk of hip fracture is about 10 times higher in a hospital setting as compared to age-matched adults in the community [20]. An IPHF rate of 0.9/1000 hospital admissions of older people has been reported [21]. A higher incidence of inpatient fall has also been reported from single rooms [5,6,7,8], particularly in those with dementia [10,22]. Although following quality initiatives the fall rate has been lower [14,17], the long-term impact of such intervention has not been reported. 

The National Hip Fracture Data (NHFD) report from 2016 reported that 3.9% (2511) patients sustained IPHF in 2015 [18]. Hip fractures which were sustained as an inpatient in various hospitals in England and Wales ranged from 0.0% to 12.5% [18]. However inter-hospital comparisons may be misleading because this variation will reflect case-mix variation in different wards, hospitals and trusts. The mean incidence of hip fracture in this study over 5 years was 2.7% (range 2.0–4.0%). Although a downward trend in IPHF was observed, further quality initiatives would be needed for further reductions. NAIF has recommended that all hospitals in the UK should monitor the effectiveness of local initiatives to prevent inpatient falls and IPHF [18]. 

Mortality following hip fracture in the community had been reported as 30% [23,24]. In this study, overall one-year mortality (2012–2016) following an inpatient fall was 46.4% and one-year mortality following IPHF was 56.5%. In a retrospective observational study comparing clinical outcomes following hip fracture in the hospital and community, one-year mortality for IPHF was 50% [25] which is similar to what we have observed. The majority of hip fractures after falls occur in medical or geriatric wards with multiple co-morbidities, but the older people admitted to psychiatric wards were at highest risk of inpatient fall and IPHF [25]. 

The National Institute for Health and Care Excellence (NICE) has provided evidence and a recommendation on the assessment and prevention of falls in older people admitted to hospital, and does not support a fall risk-prediction tool [26]. The first NAIF against the NICE guideline on falls assessment and prevention reported that there are pockets of really good care but that many hospitals are not doing everything they can to prevent falls [5]. The key findings included that 84% of patients did not have their lying and standing blood pressure recorded, almost one-third of patients using walking aids could not safely access them, limiting the ability to mobilise safely, and almost one-fifth of patients were unable to access their call bell [5]. 

Quality initiatives not only minimise fall incidents significantly, but have shown some longevity benefits. The observed one-year mortality reduction supports further strengthening and wider application of these quality interventions. In this study, we have also observed significantly higher inpatient mortality for those who had sustained recurrent inpatient fall and multiple falls in a hospital could be one of the factors that can be associated with higher inpatient mortality. Therefore, all hospitalized patients should have a prompt comprehensive multifactorial fall risk assessment following hospital admission to improve clinical outcomes. All patients should be closely monitored throughout the hospital stay, and the falls assessment should be repeated as appropriate or when there is a change in a patient’s clinical condition. Furthermore, based on the results of this study, the quality improvement projects using the PDSA methodology around inpatient falls should be implemented to ensure the sustainability of interventions and target new areas for improvement [15,27]. Diurnal and seasonal patterns in presentations with hip fractures have also been reported among people who sustained their hip fracture in a hospital (*n* = 2761) or in a care home (*n* = 12,141) [28]. Therefore, we also propose studying the pattern of inpatient falls in a hospital site as a quality-improvement initiative. NHFD has developed online charts to provide ‘live’ inpatient hip-fracture data to hospital sites in the UK that can be used to monitor the effectiveness of local initiatives to prevent inpatient falls [29].

In this study, it was observed that the number of inpatients who sustained a fall remained same over the last five years. In other words, recurrent falls were prevented rather than a ‘first inpatient fall’. In addition, more than two-thirds (70%) of patients sustained a hip fracture following a ‘first inpatient fall’ and a fracture occurred from 1 to 86 days after admission (mean days were 18.5). Falls were more common prior to discharge, as patients had regained good functional activity and physical mobility and were often waiting to be discharged. Another study reported median time to hip fracture following admission to hospital as 9.5 days and more than half of patients sustained IPHF when they already had a planned date of discharge [30]. Based on our results, if we have to prevent IPHF, we must avoid the ‘first inpatient fall’. 

Our study has several strengths. Firstly, we are not aware of any study that has reported the comprehensive clinical outcome data for inpatient falls from a hospital with 100% single rooms over 5 years. We achieved a complete follow-up for inpatient falls and mortality up to a minimum of one year. Furthermore, we have attempted to measure the long-term impact of quality initiatives on IPHF and mortality. This study could also help to set up some benchmark clinical outcomes to enhance patient safety and quality in the hospital setting. 

We acknowledge methodological weaknesses. This was an observational study based on incident reporting and we have only analysed clinical outcome data. We also acknowledge that this is a single-centre study and we have not studied many complex patient characteristics such as the reason for admission, co-morbidities, dementia, incontinence or polypharmacy. A major limitation of this retrospective observation study to measure the exact impact of quality initiatives to minimize inpatient falls and prevent adverse clinical outcomes remains the lack of control group, to demonstrate that the incidence of falls reduction is not secondary to unmeasured confounders or even just a chance finding. 

The observed incidence of inpatient falls, associated injury and high mortality will certainly make managing frail older people in hospitals more challenging, suggesting further research to support policy-making and quality of care. A comprehensive multifactorial fall risk assessment and a targeted multidisciplinary falls action plan could minimise the incidence of hip fractures in hospitalized patients. Furthermore, investment in existing intermediate care services, strengthening links with primary care, liaising with social services, and moving care closer to a patient’s home could avoid delays in the transfer of care. This will perhaps minimise the incidence of inpatient falls that has actually been observed closer to discharge date. 

## 5. Conclusions

We have observed a significant reduction in the incidence of inpatient falls following quality initiatives initially, followed by a downward trend in the rate of hip fractures. We have just started to observe a significant reduction in the 6-month and one-year mortality rates in two successive years (2015–2016) following implementation of the quality initiatives. Prompt completion of multifactorial falls risk assessments and every possible quality initiative should be taken to avoid a ‘first in-patient fall’ which will surely result in sustained improved clinical outcomes. The timely and prompt transfer of care from hospital to the community should be encouraged to avoid inpatient falls closer to a discharge date.
